# An alternate hybrid PWM for uniform thermal sharing in single phase voltage-source inverter

**DOI:** 10.1038/s41598-023-30169-y

**Published:** 2023-02-27

**Authors:** K. S. P. Kiranmai, R. V. Damodaran, M. Hushki, H. Shareef

**Affiliations:** 1 Department of Electrical and Communication Engineering, United Arab Emirates University, PO Box 15551, Al Ain, United Arab Emirates; 2 National Water and Energy Center, UAE University, PO Box 15551, Al Ain, United Arab Emirates

**Keywords:** Electrical and electronic engineering, Energy infrastructure, Energy science and technology

## Abstract

The most prevalent reason for IGBT failure in voltage-source inverter (VSI) is thermal stress, which is influenced by the topology and modulation technique adopted. Hence, it is crucial to understand the impact of common sinusoidal pulse width modulation (PWM) strategies on the thermal behavior of VSI and develop improved PWM techniques. This paper presents an alternate hybrid PWM (AHPWM) switching sequence for a single-phase VSI to decrease its thermal stress and prolong its lifetime. To evaluate the number of cycles to failure of VSI, power loss and thermal analysis were conducted for VSI with AHPWM and compared with conventional PWMs. The thermal cycles experienced by IGBTs with different modulation techniques were experimentally validated using a laboratory prototype of VSI. These findings suggest that by employing AHPWM, the likelihood of VSI failure is reduced by at least one-and-a-half times, resulting in lower VSI maintenance costs.

## Introduction

A single-phase full-bridge voltage-source inverter (VSI) is a common power electronic converter employed in applications where DC-to-AC conversion is required. Its applications range from electric vehicles, microgrids, and solar power systems to industrial applications such as speed drives^1–7^. VSI is an important element in determining the reliability of a system. However, VSIs are frequently prone to failure because of their critical components, such as capacitors and semiconductor power devices^8–10^. Semiconductor switches, which are essentially insulated gate bipolar transistors (IGBT), contain several semiconductor dies within a small footprint bonded to substrates with aluminum wires and wide-area solder joints. During operation, IGBTs are subjected to periodic variations in their junction temperatures, referred to as thermal cycling^11^. High-temperature swings at high frequencies cause mechanical fatigue due to expansions and contractions. This can break the connection between the dies and bond wires, significantly reducing the lifespan of the switches. Hence, the analysis of IGBT thermal cycles is important for predicting the failure time^12–15^.

The amplitude and frequency of the thermal cycles experienced by an IGBT result from its power loss, which comprises conduction, switching, and blocking losses. Because of their small magnitudes, blocking losses can be neglected. Switching losses are influenced by the IGBT switching frequency, whereas conduction losses are determined by the load current. In a single-phase VSI, the total power loss at any given time is the sum of the power losses of the active switches. The number of active switches at a given time, and their voltage and current stress are determined by pulse-width modulation (PWM) methods^16^. As a result, PWM approaches are critical in influencing the total power loss, and thus the thermal stress in a VSI.

Sinusoidal PWM techniques, such as bipolar PWM (BPWM), unipolar PWM (UPWM), and hybrid PWM (HPWM), are widely used for single-phase VSIs because of their ease of implementation^17^. Currently, the parameters that determine the PWM technique selection are efficiency and power quality. The effects of PWM techniques on inverter efficiency and total harmonic distortion (THD) were investigated in^18–20^, while the effects of PWM on the current ripple for UPWM and BPWM were investigated in^21^. In^22^, Gupta et al. presented a novel PWM technique for reducing THD by increasing the switching frequency. In addition to the efficiency and power quality, PWM techniques affect the thermal stresses of IGBTs in VSI. Thermal analyses of single-phase VSI under different climatic conditions, topologies, and power sources were presented in^10,23,24^. Similarly, in^25^, the effects of UPWM and BPWM on the lifetime of a DC-link capacitor were discussed. IGBTs are the most sensitive of the constituent components of VSI; hence, performing a thermal analysis of the VSI for various PWM approaches is essential.

The thermal behavior of a VSI is affected by the number of active switches, the switching frequency, and the duration of operation. The BPWM and UPWM techniques require all four switches to operate at high frequencies, resulting in a uniform but high switching loss and thermal stress. On the other hand, in HPWM, two switches in one leg operate at a high frequency, while the two switches in the other leg operate at the system frequency. The four switches have a non-uniform thermal stress distribution, with the fast switches subjected to high power losses and slow switches subjected to low power losses. Nonetheless, because VSI failure is determined by the switch with the highest thermal stresses, HPWM has a failure rate comparable to BPWM and UPWM. To extend the time to VSI failure, it is vital to maintain uniform and minimized thermal sharing among the switches. Several bus clamping techniques can be found in the existing literature for three-phase VSI that can achieve uniform loading of the switches^26,27^. A few articles discuss bus clamping techniques for single-phase VSI^28^ that ensure uniform loading of the switches, but at the expense of the increased complexity of modulating signal generation. Furthermore, the selection of a bus-clamping technique is based on a trade-off between the switching loss, equal loading, and THD.

To improve the number of cycles to failure of VSI, a novel switching sequence called alternate hybrid PWM (AHPWM) to control VSI is proposed in this paper. It reduces power losses, thus improving the efficiency, superior thermal performance, and improved lifespan of the VSI. The major contributions of this study are as follows.

- Conduct a power loss analysis of VSI for the proposed AHPWM, BPWM, UPWM, and HPWM.
- Perform thermal analysis of VSI in PLECS simulator.
- Estimate the number of cycles to failure using Coffin–Manson law and rainflow algorithm.
- Carry out experiments to verify the thermal cycles for different PWMs.

## Methods

### Alternate hybrid PWM technique for uniform thermal sharing

**Figure 1:**
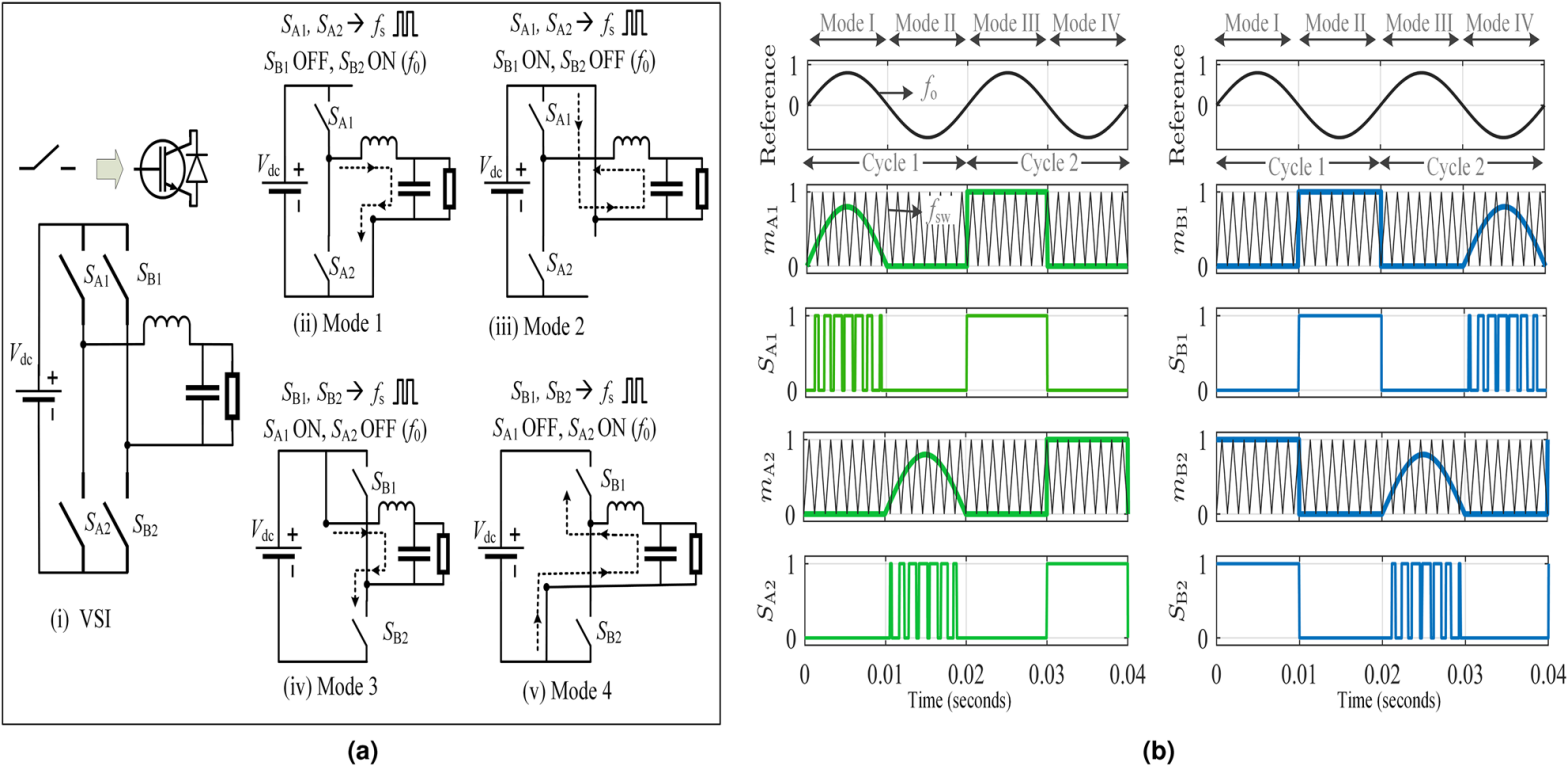
(**a**) Single phase VSI and its operating modes with AHPWM, (**b**) sinusoidal reference, modulating signal, carrier and resulting switching pulses for the proposed AHPWM.

A circuit diagram of a single-phase VSI connected to a load through an LC filter is shown in Fig. 1a(i)). It consists of two legs, namely A and B. Leg A consists of switches $S_{\textrm{A1}}$ and $S_{\textrm{A2}}$, while Leg B comprises switches $S_{\textrm{B1}}$ and $S_{\textrm{B2}}$. To ensure consistent thermal distribution among the four switches of the VSI, a modulation technique called alternate hybrid PWM (AHPWM) derived from HPWM is proposed in this paper. This modulation technique effectively reduces the magnitude and frequency of the thermal stress, improving the thermal performance while retaining the electrical characteristics of the VSI as HPWM.

The AHPWM cycle comprises four operating modes spanning two full reference cycles. The circuit operation during these four modes is shown in Fig. 1a(ii)–(v)). The switching pulses for the four switches were obtained by comparing four different modulating signals derived from a sinusoidal reference waveform of frequency $f_{\textrm{o}}$ with a carrier signal of switching frequency $f_{\textrm{sw}}$. The modulating signal and the resulting switching pulses for the switches of the VSI during the two reference cycles are shown in Fig. 1b. The four operating modes are discussed below.

*Mode I* ($0\le t<0.01$): The VSI operates in mode I during the positive half cycle of the first reference cycle. In this mode, the switch $S_{\textrm{A1}}$ is pulse width modulated at $f_{\textrm{sw}}$ while $S_{\textrm{B2}}$ remains ON throughout the half cycle.

*Mode II* ($0.01\le t<0.02$): In the negative half cycle of the first reference cycle, the switch $S_{\textrm{A2}}$ is pulse width modulated at $f_{\textrm{sw}}$ while $S_{\textrm{B1}}$ remains ON.

*Mode III* ($0.02\le t<0.03$): In this mode, the VSI operates in the positive half cycle of the second reference cycle. Switches $S_{\textrm{B2}}$ and $S_{\textrm{A1}}$ operate at $f_{\textrm{sw}}$ and $f_{\textrm{o}}$, respectively, during this period.

*Mode IV* ($0.03\le t<0.04$): The circuit operates in this mode during the negative half cycle of the second reference cycle. In this mode, switches $S_{\textrm{B1}}$ and $S_{\textrm{A2}}$ are operated at $f_{\textrm{sw}}$ and $f_{\textrm{o}}$, respectively.

In summary, the operating frequency of the switches alternate between $f_{\textrm{sw}}$ and $f_{\textrm{o}}$ during the two reference cycles. Hence, each switch operates at $f_{\textrm{sw}}$ only once during the two-reference cycle period. As a result, all four switches have significantly less switching power loss, and they share the thermal stress equally, thereby improving the number of cycles to failure of the VSI.

### Power loss estimation

The power loss in switching devices must be investigated to understand their thermal behavior^16^, as this is the cause of the increase in thermal stress in the switch. The total power loss in a VSI at any given time is the sum of the power losses in all switches operating at that instant. The IGBT switch used in this work consists of an IGBT and a free-wheeling diode (FWD), contributing to losses $P_\mathrm {total\_S}$ and $P_\mathrm {total\_D}$ respectively. The total power loss is obtained from (1):

1 $$\begin{aligned} P_\mathrm{total}=P_\mathrm {total\_S}+P_\mathrm {total\_D} \end{aligned}$$

The IGBT power loss $P_\mathrm {total\_S}$ consists of conduction losses $P_\mathrm {cond\_S}$ and switching losses $P_\mathrm {sw\_S}$ as shown in (2).

2 $$\begin{aligned} P_\mathrm {total\_S}&=P_\mathrm {sw\_S}+P_\mathrm {cond\_S} \end{aligned}$$

3 $$\begin{aligned} \text {where }P_\mathrm {cond\_S}&=v_\mathrm{ceo}i_\mathrm{c}(t)+r_\mathrm{c}i_\mathrm{c}^2(t) \end{aligned}$$

4 $$\begin{aligned} \text {and }P_\mathrm {sw\_S}&=(E_\mathrm {on\_S}+E_\mathrm {off\_S})f_{\textrm{sw}} \end{aligned}$$

In (3), $v_\mathrm{ceo}$ is the collector emitter voltage at the on-state zero current, $r_\mathrm{c}$ is the collector emitter on state resistor, and $i_\mathrm{c}$ is the collector current. In the (4), $E_\mathrm {on\_S}$ and $E_\mathrm {off\_S}$ are the turn-on and turn-off switching energy losses of the IGBT, respectively, and $f_{\textrm{sw}}$ is the switching frequency. The diode power loss $P_\mathrm {total\_D}$ consists of conduction loss $P_\mathrm {cond\_D}$ and switching loss $P_\mathrm {sw\_D}$ given by (5) and (6), respectively.

5 $$\begin{aligned} P_\mathrm {cond\_D}=v_\mathrm{Do}i_\mathrm{D}(t)+r_\mathrm{D}i_\mathrm{D}^2(t) \end{aligned}$$

where $v_\mathrm{Do}$ is the voltage across the diode at on-state zero current, $i_\mathrm{D}$ is the current through the diode, and $r_\mathrm{D}$ is the on-state resistance of the diode. $r_\mathrm{D}$ can be obtained from the data sheet of the IGBT^29^.

6 $$\begin{aligned} P_\mathrm {sw\_D}=E_\mathrm {on\_D}f_{\textrm{sw}}, \end{aligned}$$

where $E_\mathrm {on\_D}$ is the turn-on energy loss of the diode. Normally, in a diode, turn-off losses are assumed to be zero.

From (3), (4), (5), and (6), the total power loss in an IGBT switch is a function of the switching frequency $f_{\textrm{sw}}$ and the current through the switch. The VSI power losses in the AHPWM approach were analyzed by varying these factors.

### Thermal modelling

**Figure 2:**
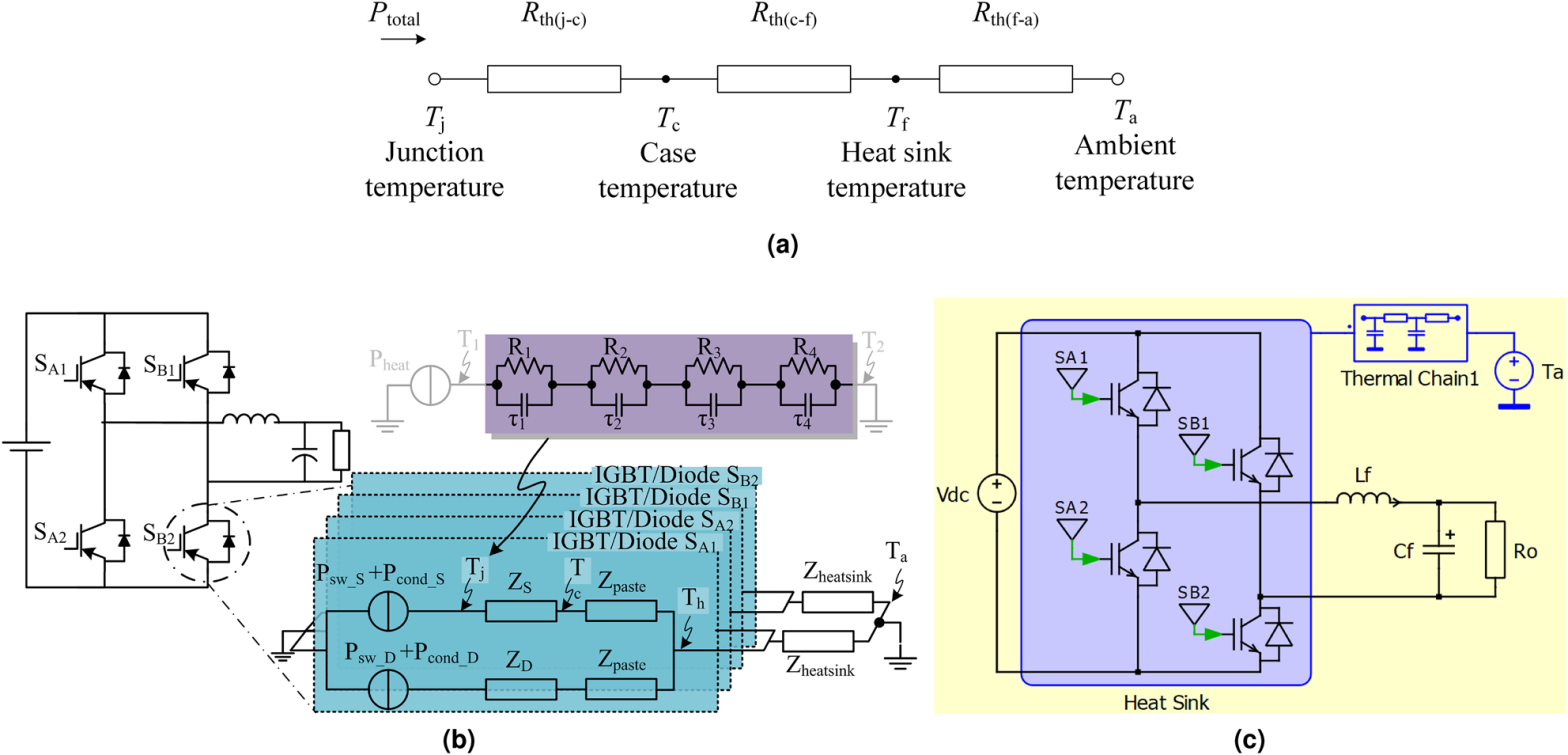
(**a**) Thermal equivalent circuit, (**b**) thermal modelling of VSI and (**c**) PLECS model for thermal analysis of VSI.

The switch power loss is dissipated through the heatsink to the ambient air and can be represented using a thermal resistance equivalent circuit, as shown in Fig. 2a. The junction temperature of the switches increases in proportion to the power loss^30^ and can be defined as

7 $$\begin{aligned} T_\mathrm{j}=R_\mathrm {th(j-c)}\cdot P_\mathrm{total}+T_\mathrm{C} \end{aligned}$$

where $R_\mathrm {th(j-c)}$ is the thermal impedance from junction to case and $T_\mathrm{C}$ is the case temperature. This in turn is defined as

8 $$\begin{aligned} T_\mathrm{C}=T_\mathrm{a}+(R_\mathrm {th(c-f)}+R_\mathrm {th(f-a)})\cdot P_\mathrm{total} \end{aligned}$$

where $T_\mathrm{a}$ is the ambient temperature, and $R_\mathrm {th(c-f)}$ and $R_\mathrm {th(f-a)}$ are the thermal impedances from the case to the heat sink and from the heat sink to the ambient, respectively.

The thermal model of the VSI presented in Fig. 2b represents all four IGBT thermal resistance circuits connected to ambient temperature through a heat sink. The thermal resistance from the junction to the case of the IGBT is represented using the Foster network model, which is a widely used thermal model. The junction temperature of each switch is directly proportional to the power dissipated in the switch, as well as $R_\mathrm {th(j-c)}$, $R_\mathrm {th(c-f)}$ and $R_\mathrm {th(f-a)}$. Because the thermal parameters are constant, the thermal behavior is directly affected by the power dissipated through the switch.

#### Thermal model of VSI in PLECS

To determine the IGBT power loss and thermal analysis, a thermal model of the VSI was developed in parallel with the electrical model using PLECS, a power electronics simulation platform in MATLAB, as shown in Fig. 2c. PLECS allows users to provide the necessary parameters for the loss estimation of power semiconductors. These parameters include the threshold voltage, plateau voltage, and the Foster and Cauer thermal resistor-capacitor networks. These are often provided by the device manufacturer and can be used for the numerical estimation of losses using equations or implemented in the form of lookup tables. The electrical model estimates the losses of the power semiconductors as a function of interdependent parameters based on (9).

9 $$\begin{aligned} P_\mathrm{total}=f(I_\mathrm{C},V_\mathrm{CE},R_\mathrm{g},T_\mathrm{j},f_{\textrm{sw}}) \end{aligned}$$

where $I_\mathrm{C},V_\mathrm{CE},R_\mathrm{g},T_\mathrm{j}$, and $f_{\textrm{sw}}$ represent the collector current, collector-to-emitter voltage, on-state resistance, junction temperature, and switching frequency of the IGBT, respectively. The calculated power losses were then translated into thermal cycles using the thermal model.

Thermal cycles are graphical representations of the junction temperature $T_\mathrm{j}$ and are dependent on the power cycles to which the device is subjected. These cycles are characterized by their amplitude ($\Delta T_\mathrm{j}$), maximum value ($T_\mathrm{jmax}$), and frequency. For a VSI, the frequency of the thermal cycles is the same as the system frequency ($f_{\textrm{o}}$). It is challenging to measure $\Delta T_\mathrm{j}$ and $T_\mathrm{jmax}$ while the switch is in operation. Hence, a parameter termed the virtual junction temperature, $T_\mathrm{vj}$, is defined with amplitude $\Delta T_\mathrm{vj}$ and maximum value $T_{\textrm{vjmax}}$ that can be determined using PLECS.

### Estimation of number of cycles to failure

Lifetime models were used to determine the impact of $\Delta T_\mathrm{vj}$ and $T_{\textrm{vjmax}}$ on each IGBT lifespan and to estimate the total lifetime of the VSI. The Coffin–Manson law is the most widely used lifetime model. It calculates the mean value of the number of cycles to failure ($N_\mathrm{f}$) as a function of junction temperature, as shown in (10)^30–32^.

10 $$\begin{aligned} N_\mathrm{f}=A(\Delta T_\mathrm{vj})^{-\alpha }e^{\left( \frac{E_\mathrm{a}}{K_\mathrm {\beta }T_{\textrm{vjmax}}}\right) } \end{aligned}$$

where *A* = Technology coefficient, $\alpha$ = Coffin–Manson exponent, $E_\mathrm{a}$ = activation energy, $K_\mathrm {\beta }$ = Boltzmann constant.

The values of *A* and $\alpha$ are obtained using the curve-fitting technique from the power-cycling curves in^33^.

**Figure 3:**
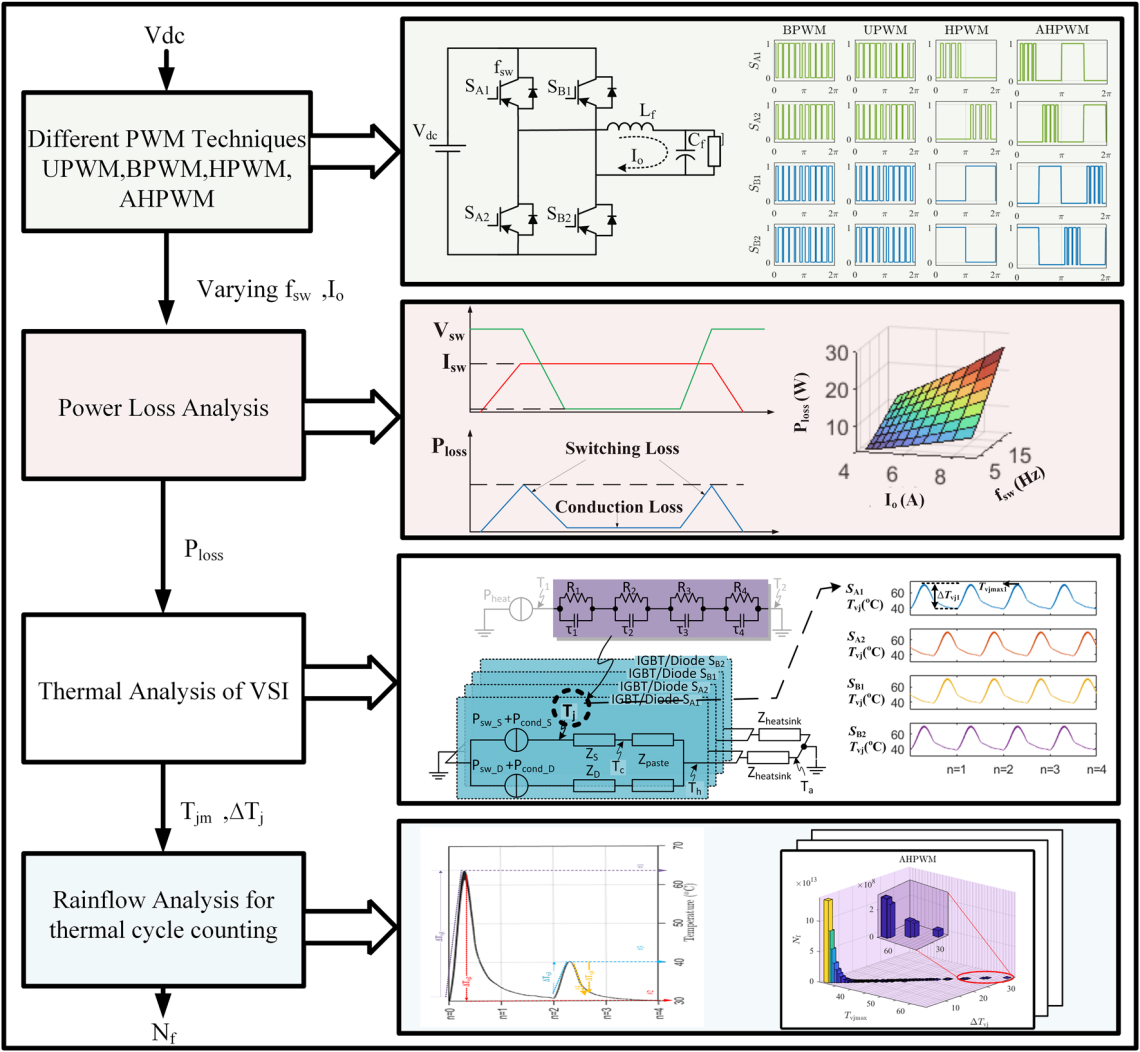
Flow diagram for estimating the number of cycles to failure of VSI.

Figure 3 shows the approach for estimating the number of cycles to VSI failure, $N_\mathrm{f}$ for AHPWM in comparison with BPWM, UPWM, and HPWM.

## Results and discussion

In this section, the power loss and thermal analysis of a single-phase VSI were carried out for the proposed AHPWM and compared with those of different PWM techniques. A resistive load was connected to the output of VSI through an LC filter, and IGBT was used as the switching device. The specifications of the VSI are given in Table 1.

**Table 1:** System specification for comparison of PWM techniques.

Parameter	Value
Rated power	2 kW
Input voltage ($V_{\textrm{in}}$ )	330 V
Output voltage ($V_{\textrm{o}}$ )	220 V
Output frequency ($f_{\textrm{o}}$)	50 Hz
Switching frequency ($f_{\textrm{sw}}$ )	20 kHz
Dead time ($t_{\textrm{dt}}$)	1 $\upmu$s
Filter parameters	5 mH, 15 $\upmu$F

### Reduced and even IGBT power losses

**Figure 4:**
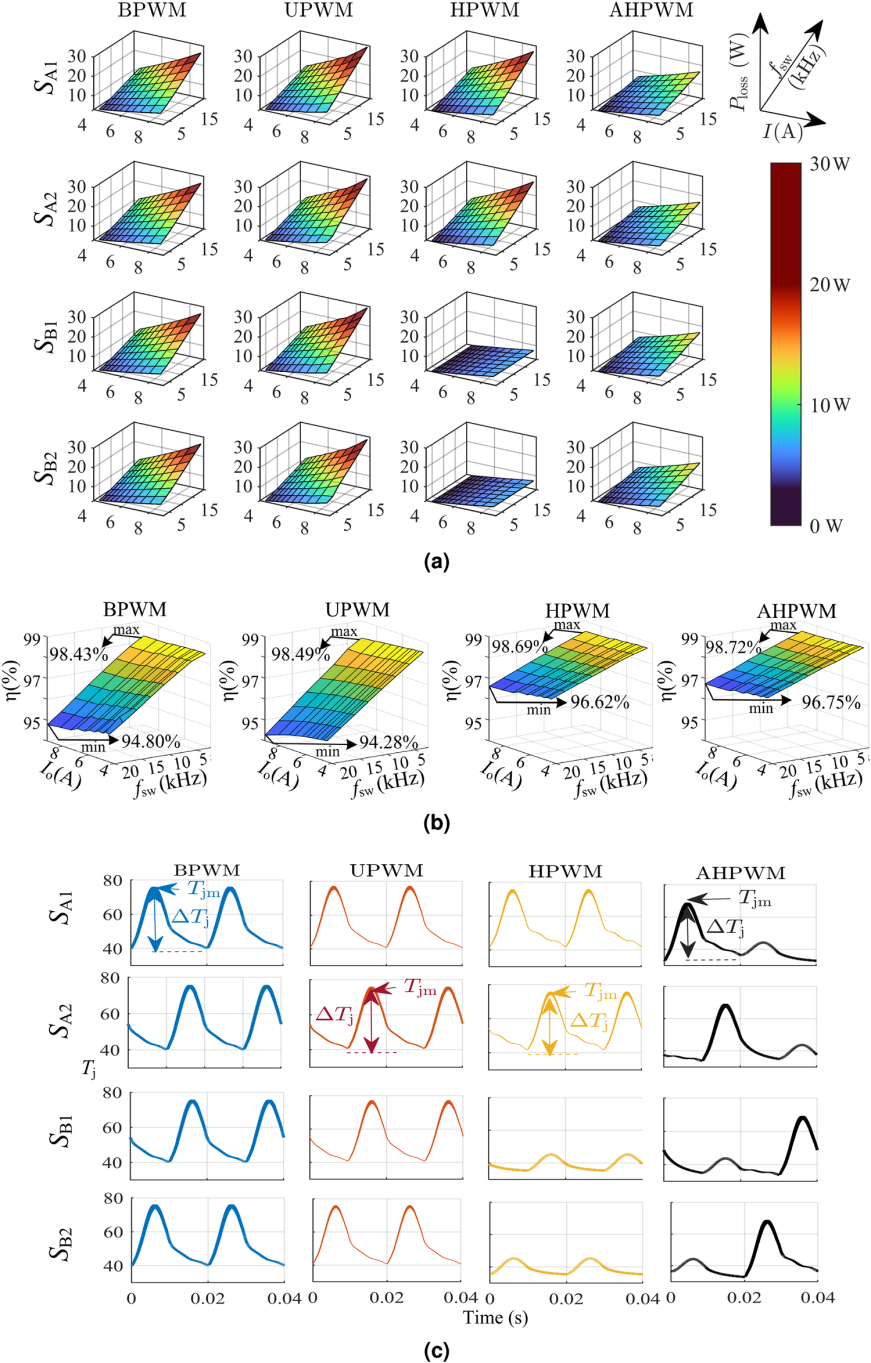
Comparison of PWMs based on (**a**) power loss, (**b**) efficiency, and (**c**) thermal cycles of VSI.

The switching losses increase significantly as the current and switching frequency increase, as per (3)–(6). Different PWM methods change the effective switching frequency and load current, consequently altering the semiconductor losses. A comparison of power losses in switches for BPWM, UPWM, HPWM, and the proposed AHPWM obtained for the same inverter configuration and rating with load current variation from 50 to 100% and $f_{\textrm{sw}}$ from 2.5  to 20 kHz is shown in Fig.4a. For BPWM and UPWM, the power losses are observed to increase with $f_{\textrm{sw}}$ and *I* and reach a maximum of 26 W for all four switches. However, in the case of HPWM, the power losses of switches $S_{\textrm{A1}}$ and $S_{\textrm{A2}}$ are similar to that of BPWM and UPWM while switches $S_{\textrm{B1}}$ and $S_{\textrm{B2}}$ experience a maximum power loss of 8 W. In the case of AHPWM, the power losses experienced by all switches are equal, with a maximum value of 16 W. The power losses vary depending on the modulation technique used. In comparison, the AHPWM method reduces and evenly distributes power losses among the four switches. In Fig. 4b, the efficiency of the VSI for the four PWM techniques at different load currents and switching frequencies is presented. The efficiency of AHPWM is observed to be the greatest under all operating conditions, varying from 96.75 to 98.72%.

### Uniform thermal cycles with reduced frequency and amplitude

The thermal cycles experienced by IGBTs in a VSI with AHPWM are compared to those of BPWM, UPWM, and HPWM for $f_{\textrm{sw}}$ = 20 kHz at full load and are presented in Fig. 4c. The thermal cycles are in agreement with the power losses encountered by the switches, as shown in Fig. 4a. For BPWM and UPWM, all four IGBTs have identical and high thermal stresses [$T_{\textrm{vjmax}}$, $\Delta T_\mathrm{vj}$] of [66 $^{\circ }$C, 33.5 $^{\circ }$C], and [72 $^{\circ }$C, 36 $^{\circ }$C], respectively. The frequency of the thermal cycles experienced by each switch in BPWM and UPWM is the same as the reference cycle frequency $f_{\textrm{o}}$. In HPWM, however, the two IGBTs in leg A working at $f_{\textrm{sw}}$ ($S_{\textrm{A1}}$ and $S_{\textrm{A2}}$) have high thermal stress with [67 $^{\circ }$C, 33 $^{\circ }$C], similar to that of BPWM and UPWM, but those in leg B operating at $f_{\textrm{o}}$ ($S_{\textrm{B1}}$ and $S_{\textrm{B2}}$) have a minimal stress of [39.5 $^{\circ }$C, 9 $^{\circ }$C]. Consequently, when HPWM is used, the thermal cycles experienced by the IGBTs are not uniform. However, in the case of AHPWM, all switches undergo the same thermal cycles of [63 $^{\circ }$C, 33 $^{\circ }$C]. Furthermore, because each switch alternates between $f_{\textrm{sw}}$ and $f_{\textrm{o}}$, the effective thermal cycle frequency is reduced by half, that is, $\frac{f_{\textrm{o}}}{2}$.

**Figure 5:**
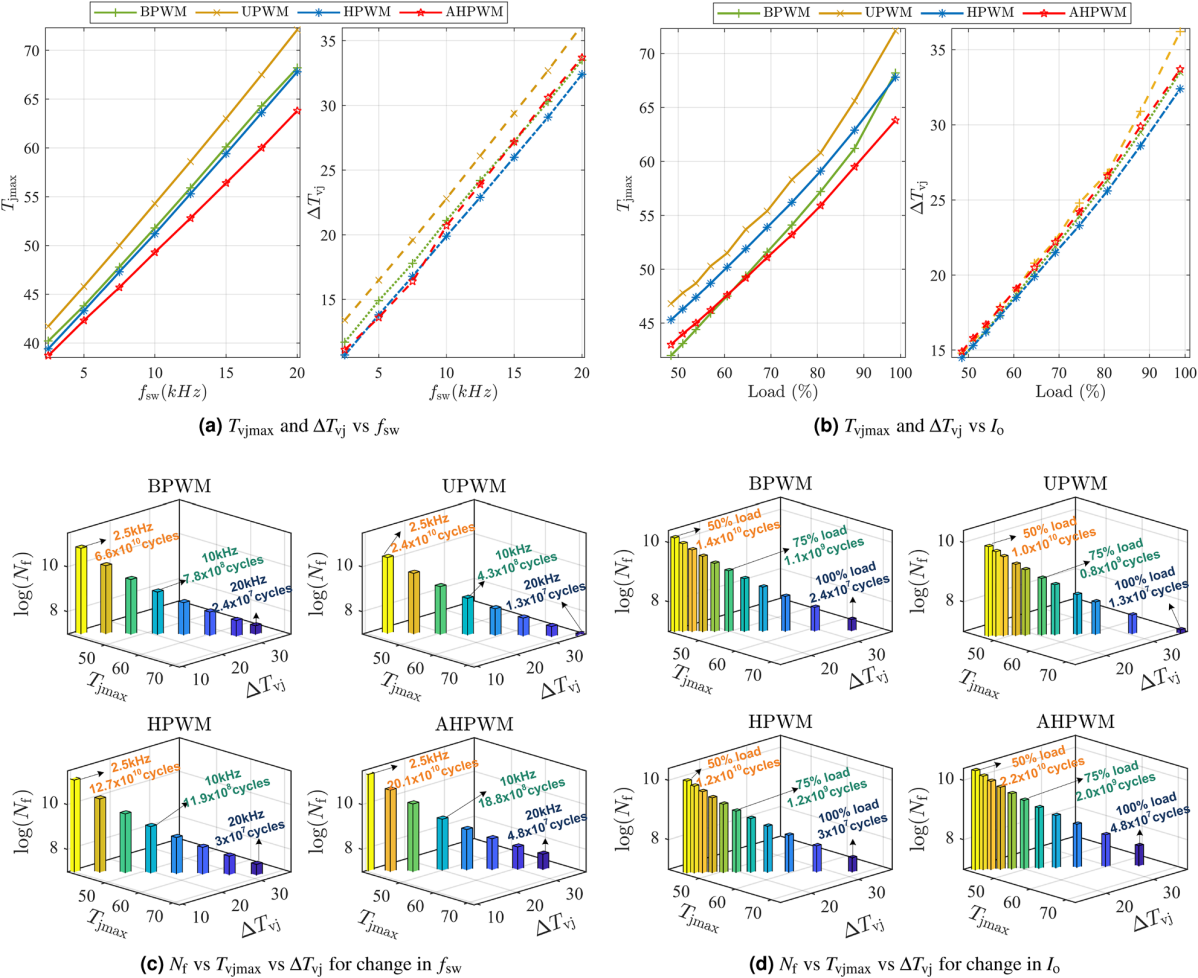
Thermal behavior and number of cycles to failure of VSI for different PWMs with varying $f_{\textrm{sw}}$ and $I_{\textrm{o}}$.

In Fig. 5a,b, the effects of the change in $f_{\textrm{sw}}$ and load percentage on the thermal cycles of the VSI are compared for various PWMs.

- Case study 1: Effect of change in switching frequency
  In this case, the VSI was operated at full load and the thermal cycles were observed by varying $f_{\textrm{sw}}$ from 2.5  to 20 kHz in steps of 2.5 kHz. The values of $T_{\textrm{vjmax}}$ and $\Delta T_\mathrm{vj}$ for different PWMs are presented in Fig. 5a. Both $T_{\textrm{vjmax}}$ and $\Delta T_\mathrm{vj}$ are observed to increase with frequency for all the PWMs. For BPWM, UPWM, HPWM and AHPWM, the values of $T_{\textrm{vjmax}}$ increase from 40 to 66 $^{\circ }$C, 42 to 72 $^{\circ }$C, 39 to 67 $^{\circ }$C and 38 to 63 $^{\circ }$C, respectively, as the frequency varies from 2.5 to 20 kHz. It is notable that amongst the four PWMs, the values of $T_{\textrm{vjmax}}$ are the lowest in the case of AHPWM for all frequencies. The gap between the values of $T_\mathrm{vj}$ for AHPWM and other PWMs widens as the frequency increases. However, values of $\Delta T_\mathrm{vj}$ for all the PWMs are comparable and vary between approximately 11 and 36 $^{\circ }$C.
- Case study 2: Effect of change in load
  In this case, the VSI was operated at $f_{\textrm{sw}}=20$ kHz while the load changed from $50\%$ to full load. As the load increases, $T_{\textrm{vjmax}}$ and $\Delta T_\mathrm{vj}$ also increase as shown in Fig. 5b. The values of $T_\mathrm{vj}$ are 42 $^{\circ }$C, 47 $^{\circ }$C, 45 $^{\circ }$C and 43 $^{\circ }$C at $50\%$ load for BPWM, UPWM, HPWM and AHPWM and increase to 66 $^{\circ }$C, 72 $^{\circ }$C, 67 $^{\circ }$C and 63 $^{\circ }$C, respectively, at full load. From these values, it can be seen that AHPWM has comparably lower $T_\mathrm{vj}$ values, in particular at higher loading conditions. As with the previous case, $\Delta T_\mathrm{vj}$ is similar for all the PWMs and varies between 14 and 36 $^{\circ }$C.

**Table 2:** $N_\mathrm{f}$ of AHPWM in comparison with other PWMs for different switching frequencies and loads.

$f_{\textrm{sw}}$ (kHz)	BPWM (%)	UPWM (%)	HPWM (%)	Load (%)	BPWM (%)	UPWM (%)	HPWM (%)
2.5	300	840	160	50	160	220	180
10	240	440	160	75	180	250	160
20	200	360	160	100	200	360	160

In the above two case studies, AHPWM is observed to have a reduced frequency and amplitude for the thermal cycles in comparison to other PWMs which implies reduced thermal stress.

### Enhanced number of cycles to failure

The effect of uniform temperature cycles with reduced amplitude and frequency on the longevity of the switches is examined in this section. Rainflow cycle counting method^32,34,35^ was used for irregular temperature profiles of AHPWM. For a comparative evaluation, the number of cycles to failure $N_\mathrm{f}$ was obtained for two case studies, and the improvement in $N_\mathrm{f}$ when using AHPWM compared to the other three conventional PWMs is presented in terms of the percentage increase.

- Case study 1: Effect of change in switching frequency
  As shown in Fig. 5c, $N_\mathrm{f}$ for each frequency is plotted against the corresponding $T_{\textrm{vjmax}}$ and $\Delta T_\mathrm{vj}$. Switching losses increase when $f_{\textrm{sw}}$ rises, raising $\Delta T_\mathrm{vj}$ and $T_{\textrm{vjmax}}$ and lowering $N_\mathrm{f}$ for all four PWMs. The $N_\mathrm{f}$ achieved with AHPWM is higher for all of the frequencies investigated. For example, at 20 kHz, AHPWM has a $N_\mathrm{f}$ of 4.8$\times 10^7$, whereas BPWM has 2.4$\times 10^7$, UPWM has 1.3$\times 10^7$, and HPWM has 3$\times 10^7$.
- Case study 2: Effect of change in load
  In Fig. 5d, $N_\mathrm{f}$ is shown for different load currents. The power losses in the switches rise as the load current increases, causing severe thermal stress and a reduction in $N_\mathrm{f}$. A comparison of $N_\mathrm{f}$ for three different loading conditions is presented in Table 2. Similar to case study 1, amongst the four PWMS, AHPWM yields the highest $N_\mathrm{f}$ for all the loads considered.

From the two case studies, it can be inferred that the proposed AHPWM yields uniform temperature cycles with reduced amplitude and frequency, which enhances $N_\mathrm{f}$ by a factor of at least 1.5, compared to other PWM techniques.

### Experimental validation

**Figure 6:**
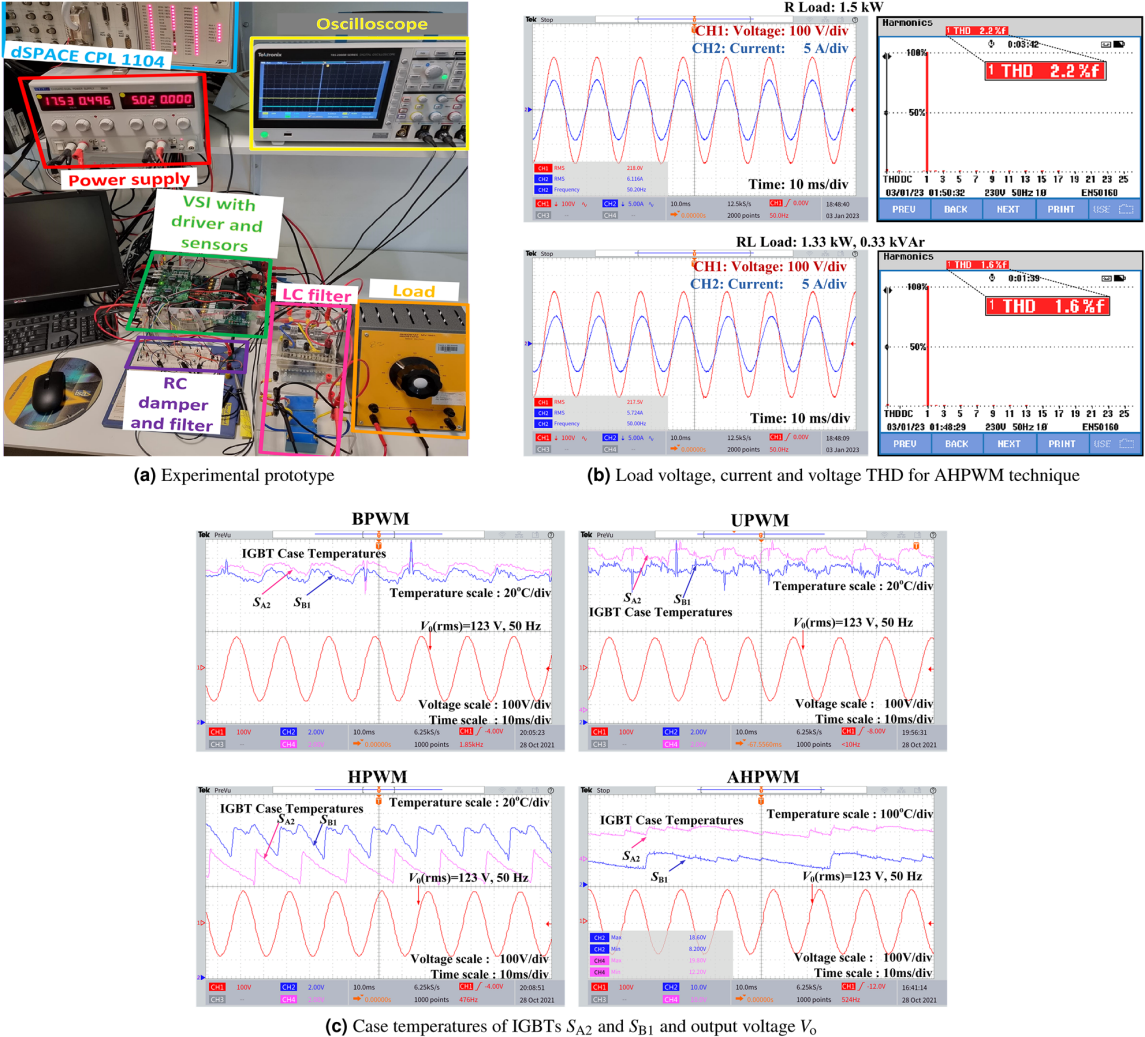
Experimental setup, results showing voltage, current and voltage THD with AHPWM and the case temperatures of IGBTs to validate the temperature variation pattern.

To validate the simulation results, an experimental prototype was developed in the laboratory as shown in Fig. 6a with the specifications listed in Table 1. The load voltage, current, and voltage THD for the proposed AHPWM technique with resistive (R) and resistive-inductive (RL) loads are shown in Fig. 6b. The R-load of 1.5 kW yields a voltage THD of 2.2%, while an RL-load of 1.33 kW and 0.33 kVAr yields a THD of 1.6%.

The temperature profiles for different PWMs were validated for the system specifications listed in Table 1 and voltages $V_\mathrm{dc}=200$ V and $V_{\textrm{o}}=120$ V. IGBT IKW15N120H3 were used as switches in the VSI. The RTI dSPACE CLP 1104 was used to implement UPWM, BPWM, HPWM, and the proposed AHPWM switching patterns. It is difficult to measure the junction temperature of IGBTs accurately using commonly used temperature sensors. Hence, in this study, the temperatures of the IGBTs were measured to validate the pattern of temperature variations. For this purpose, LM35 temperature sensors were placed on the case of the IGBTs. RC damper and RC filter circuits were used to couple the output of the LM35 sensors to the ADC channels of dSPACE^36^ and to filter out the high-frequency noise, respectively (Supplementary Information).

The output voltage $V_{\textrm{o}}$ and case temperatures of $S_{\textrm{A2}}$ and $S_{\textrm{B1}}$ for UPWM, BPWM, HPWM and the proposed AHPWM are presented in Fig. 6c. The measured thermal cycles of the IGBTs (case temperatures) for the four PWMs considered were similar in pattern to those obtained in the simulation, as shown in Fig. 4c. It can be observed from the case temperature variation of AHPWM in Fig. 6c that the switches experience the minimum number of thermal cycles when the AHPWM technique is employed. This finding validates the theoretical claims presented in this paper.

## Conclusion and future work

A novel modulation technique called AHPWM, to achieve uniform and reduced thermal sharing among switches in a single-phase VSI, is presented in this paper. The impact of different modulation techniques on the lifetime of the IGBT in a VSI was explored. The results demonstrate that the applied modulation technique influences the thermal cycles of the IGBTs used. Consequently, different modulation techniques cause distinct thermal cycles, which have varying effects on the lifespan of a VSI. The suggested AHPWM switching sequence significantly lowers the temperature swing and number of thermal cycles that each IGBT endures during operation. This can effectively improve the number of cycles to failure of the VSI. The proposed AHPWM technique will decrease the cost of VSI maintenance owing to its increased lifespan.

As part of future work, the proposed AHPWM technique will be extended to other single-phase and three-phase VSI configurations to achieve uniform and low power losses and thermal stresses among the switching devices. A comparison and study of different PWM techniques will also be conducted for other switching devices, such as wide band-gap devices.

## Supplementary Information

Supplementary Information.

## Data Availability

All data generated during this work are presented in the manuscript and in the associated supplementary file.

## References

[1] Semsar S, Soong T, Lehn PW (2020). On-board single-phase integrated electric vehicle charger with v2g functionality. IEEE Trans. Power Electron..

[2] Parvez M (2020). Comparative study of discrete pi and pr controls for single-phase ups inverter. IEEE Access.

[3] Pokharel M, Hildebrandt N, Ho CNM, He Y (2018). A fast-dynamic unipolar switching control scheme for single-phase inverters in dc microgrids. IEEE Trans. Power Electron..

[4] Viswadev R (2021). A precise switching frequency formulation of hysteresis controlled grid connected inverters considering non-linear ripple current. IEEE Trans. Ind. Electron..

[5] Lee K, Ha J-I (2016). Single-phase inverter drive for interior permanent magnet machines. IEEE Trans. Power Electron..

[6] Kumar, B. & Srinivas, S. Space vector based pwm of dual full-bridge vsi fed two-phase induction motor drive. In *2014 IEEE 23rd International Symposium on Industrial Electronics (ISIE)*, 667–672 (IEEE, 2014).

[7] Awaar, V. K., Jugge, P. *et al.* Field test of cost effective voltage source inverter for driving an induction motor. In *2015 Annual IEEE India Conference (INDICON)*, 1–6 (IEEE, 2015).

[8] Nagarajan, A., Thiagarajan, R., Repins, I. L. & Hacke, P. L. Photovoltaic inverter reliability assessment. Tech. Rep., National Renewable Energy Lab.(NREL), Golden, CO (United States) (2019).

[9] Yang S (2011). An industry-based survey of reliability in power electronic converters. IEEE Trans. Ind. Appl..

[10] Baburajan, S., Peyghami, S., Kumar, D., Blaabjerg, F. & Davari, P. Effect of unipolar and bipolar spwm on the lifetime of dc-link capacitors in single-phase voltage source inverters. In *2020 22nd European Conference on Power Electronics and Applications (EPE’20 ECCE Europe)*, p. 1 (IEEE, 2020).

[11] Reigosa PD, Wang H, Yang Y, Blaabjerg F (2015). Prediction of bond wire fatigue of igbts in a pv inverter under a long-term operation. IEEE Trans. Power Electron..

[12] Filicori F, Bianco CGL (1998). A simplified thermal analysis approach for power transistor rating in pwm-controlled dc/ac converters. IEEE Trans. Circ. Syst. I Fundam. Theory Appl..

[13] Lai W (2015). Low $$delta t_j$$ stress cycle effect in igbt power module die-attach lifetime modeling. IEEE Trans. Power Electron..

[14] Bouguerra, S., Agroui, K., Gassab, O., Sangwongwanich, A. & Blaabjerg, F. Lifetime estimation and reliability of pv inverter with multi-timescale thermal stress analysis. In *2019 International Aegean Conference on Electrical Machines and Power Electronics (ACEMP) & 2019 International Conference on Optimization of Electrical and Electronic Equipment (OPTIM)*, 402–408 (IEEE, 2019).

[15] Wang H (2013). Transitioning to physics-of-failure as a reliability driver in power electronics. IEEE J. Emerg. Sel. Top. Power Electron..

[16] Ji, B., Chen, L., Cao, W., Zhang, M. & Pickert, V. Electro-thermal method for semiconductor power losses analysis under different modulation schemes. In *7th IET International Conference on Power Electronics, Machines and Drives (PEMD 2014)* (IET, 2014).

[17] Lai R-S, Ngo KD (1995). A pwm method for reduction of switching loss in a full-bridge inverter. IEEE Trans. Power Electron..

[18] Li RT, Chung HS-H, Lau W-H, Zhou B (2010). Use of hybrid pwm and passive resonant snubber for a grid-connected csi. IEEE Trans. Power Electron..

[19] Chiasson, J., Tolbert, L. M., McKenzie, K. & Du, Z. A complete solution to the harmonic elimination problem. In *Eighteenth Annual IEEE Applied Power Electronics Conference and Exposition, 2003. APEC’03.*, vol. 1, 596–602 (IEEE, 2003).

[20] Kolar JW, Ertl H, Zach FC (1991). Influence of the modulation method on the conduction and switching losses of a pwm converter system. IEEE Trans. Ind. Appl..

[21] Gupta AK, Joshi MS, Agarwal V (2020). Novel multicarrier pwm scheme for a reconfigurable single-phase inverter to achieve manifold higher effective switching frequency. IEEE J. Emerg. Sel. Top. Power Electron..

[22] Floriani JCA (2004). Generalized analysis of current ripple in a pulsewidth modulation h-bridge converter with unipolar-bipolar switching. IEEE Power Electron. Lett..

[23] Batunlu C, Albarbar A (2015). A technique for mitigating thermal stress and extending life cycle of power electronic converters used for wind turbines. Electronics.

[24] Sangwongwanich A, Yang Y, Sera D, Blaabjerg F (2017). Lifetime evaluation of grid-connected pv inverters considering panel degradation rates and installation sites. IEEE Trans. Power Electron..

[25] Peyghami, S., Davari, P., Wang, H. & Blaabjerg, F. The impact of topology and mission profile on the reliability of boost-type converters in pv applications. In *2018 IEEE 19th Workshop on Control and Modeling for Power Electronics (COMPEL)*, 1–8 (IEEE, 2018).

[26] Bhavsar T, Narayanan G (2009). Harmonic analysis of advanced bus-clamping pwm techniques. IEEE Trans. Power Electron..

[27] C, C. U. & Rajendran, A. Bus clamping pwm for three level neutral point clamped inverters. In *2015 International Conference on Technological Advancements in Power and Energy (TAP Energy)*, 322–326. 10.1109/TAPENERGY.2015.7229639 (2015).

[28] Chowdhury, M. R. *et al.* An improved switching control technique for single-phase voltage source inverter. In *2020 IEEE International Conference on Applied Superconductivity and Electromagnetic Devices (ASEMD)*, 1–2. 10.1109/ASEMD49065.2020.9276322 (2020).

[29] Infineon Technologies AG. *High Speed Switching Series Third Generation* (2010). Rev. 1.2.

[30] Zhang, Y., Wang, H., Wang, Z., Yang, Y. & Blaabjerg, F. Impact of lifetime model selections on the reliability prediction of igbt modules in modular multilevel converters. In *2017 IEEE Energy Conversion Congress and Exposition (ECCE)*, 4202–4207 (IEEE, 2017).

[31] Due, J., Munk-Nielsen, S. & Nielsen, R. Lifetime investigation of high power igbt modules. In *Proceedings of the 2011 14th European Conference on Power Electronics and Applications*, 1–8 (IEEE, 2011).

[32] Bryant AT, Mawby PA, Palmer PR, Santi E, Hudgins JL (2008). Exploration of power device reliability using compact device models and fast electrothermal simulation. IEEE Trans. Ind. Appl..

[33] Infineon Technologies AG. *Use of Power Cycling Curves for IGBT4* (2021). Rev. 2.1.

[34] Huang H, Mawby P (2012). A lifetime estimation technique for voltage source inverters. IEEE Trans. Power Electron..

[35] GopiReddy LR, Tolbert LM, Ozpineci B, Pinto JO (2015). Rainflow algorithm-based lifetime estimation of power semiconductors in utility applications. IEEE Trans. Ind. Appl..

[36] Texas Instruments. *LM35 Precision Centigrade Temperature Sensors* (2017). Rev. H.

